# Supplementary material for: Can psychedelic use benefit meditation practice? Examining individual, psychedelic, and meditation-related factors
Source: PLoS One. 2025 Feb 12;20(2):e0310160. doi: 10.1371/journal.pone.0310160 (PMC11819602; doi:10.1371/journal.pone.0310160)
Supplement: S1 Table — (DOCX) [file pone.0310160.s001.docx]

**S1 Table. Pearson correlation coefficients between the outcome variable and each of the predictors.**

| **Variable** | ***r*** | ***95 % CI*** | | ***p*_raw_** | ***p*_FDR_** |
| --- | --- | --- | --- | --- | --- |
|  |  | ***LL*** | ***UL*** |  |  |
| ***Individual Factors*** |  |  |  |  |  |
| Age | -.01 | -.08 | .06 | .771 | .817 |
| Female | .05 | -.02 | .11 | .164 | .217 |
| Education (in years) | -.06 | -.13 | .01 | .077 | .123 |
| Life satisfaction | .13 | .06 | .19 | <.001 | <.001 |
| Life worthwhile | .13 | .07 | .20 | <.001 | <.001 |
| Openness to experience | .16 | .10 | .23 | <.001 | <.001 |
| Neuroticism | -.11 | -.17 | -.04 | .002 | .006 |
| Conscientiousness | .09 | .02 | .15 | .011 | .022 |
| Agreeableness | .15 | .09 | .22 | <.001 | <.001 |
| Extraversion | .07 | .00 | .14 | .042 | .071 |
| Alcohol use (12 months) | -.01 | -.08 | .05 | .668 | .738 |
| Tobacco use (12 months) | .09 | .03 | .16 | .006 | .012 |
| Cannabis use (12 months) | .18 | -.08 | .06 | <.001 | <.001 |
| ***Psychedelic Factors*** |  |  |  |  |  |
| Psychedelics use (12 months) | .34 | .28 | .40 | <.001 | <.001 |
| Psychedelics use (lifetime) | .15 | .08 | .21 | <.001 | <.001 |
| Psychedelics starting age | .10 | .04 | .17 | .002 | .007 |
| Adequate psychedelics framework | .20 | .13 | .26 | <.001 | <.001 |
| Trust in psychedelics context | .19 | .12 | .25 | <.001 | <.001 |
| Setting intentions during psychedelic use | .30 | .23 | .36 | <.001 | <.001 |
| Safe setting | .22 | .16 | .29 | <.001 | <.001 |
| Psilocybin | .06 | .00 | .13 | .064 | .107 |
| LSD | .08 | .02 | .15 | .013 | .025 |
| Ayahuasca | .14 | .07 | .20 | <.001 | <.001 |
| 5-MeO-DMT | .17 | .11 | .24 | <.001 | <.001 |
| N,N-DMT | .23 | .16 | .29 | <.001 | <.001 |
| Mescaline | .09 | .03 | .16 | .005 | .012 |
| Other | .10 | .04 | .17 | .003 | .008 |
| ***Meditation Factors*** |  |  |  |  |  |
| Years of regular practice | -.01 | -.08 | .05 | .695 | .752 |
| Frequency of regular practice | -.05 | -.12 | .02 | .138 | .192 |
| Meditation starting age | -.04 | -.10 | .03 | .279 | .336 |
| Adequate meditation framework | .08 | .01 | .14 | .023 | .044 |
| Retreat experience | -.17 | -.23 | -.10 | <.001 | <.001 |
| Retreat days (12 months) | -.10 | -.17 | -.03 | .003 | .009 |
| Retreat days (lifetime) | -.05 | -.12 | .01 | .128 | .192 |
| Retreat longest | -.07 | -.14 | -.01 | .031 | .056 |
| Meditation practice before psychedelic experience | .002 | -.06 | .07 | .944 | .962 |
| Burmese Vipassana | -.10 | -.16 | -.03 | .004 | .010 |
| Culadasa | -.10 | -.16 | -.03 | .004 | .010 |
| Eclectic/other | .11 | .04 | .18 | .001 | .004 |
| Goenka Vipassana | -.01 | -.07 | .06 | .826 | .858 |
| Hindu practice | .09 | .03 | .16 | .006 | .013 |
| Jhana practice | -.04 | -.10 | .03 | .287 | .338 |
| Japanese Zen | .04 | -.03 | .11 | .231 | .291 |
| Modern mindfulness | .03 | -.03 | .10 | .327 | .377 |
| Rob Burbea | -.06 | -.13 | .01 | .080 | .125 |
| Sam Harris | .07 | .01 | .14 | .034 | .060 |
| Shinzen Young | -.05 | -.12 | .02 | .141 | .192 |
| Tibetan Chagzog | .05 | -.02 | .12 | .141 | .192 |
| Thich Nhat Hahn | .02 | -.05 | .08 | .620 | .700 |
| Tibetan Tantric meditation | -.04 | -.11 | .02 | .199 | .257 |
| Western loving kindness compassion | .00 | -.07 | .07 | .980 | .980 |
| Western non-dual meditation | .04 | -.03 | .11 | .243 | .300 |
| Western Vipassana | -.05 | -.12 | .02 | .137 | .192 |

*Note.* CI = confidence interval, fdr = False discovery rate; LSD = Lysergic acid diethylamide; 5-MeO-DMT = 5-methoxy-N,N-dimethyltryptamine N,N-DMT = N,N-Dimethyltryptamine, .
